# MPG-SwinUMamba: High-Precision Segmentation and Automated Measurement of Eye Muscle Area in Live Sheep Based on Deep Learning

**DOI:** 10.3390/ani15243509

**Published:** 2025-12-05

**Authors:** Zhou Zhang, Yaojing Yue, Fuzhong Li, Leifeng Guo, Svitlana Pavlova

**Affiliations:** 1 College of Agricultural Engineering, Shanxi Agricultural University, Jinzhong 030801, China; 2 College of Software, Shanxi Agricultural University, Taiyuan 030031, China; 3 Institute of Agricultural Information, Chinese Academy of Agricultural Sciences, Beijing 100080, China; 4 Lanzhou Institute of Husbandry and Pharmaceutical Sciences, Chinese Academy of Agricultural Sciences, Lanzhou 730050, China; 5 Xinjiang Key Laboratory of Smart Animal Husbandry, Urumqi 830011, China

**Keywords:** deep learning, image segmentation, automated phenotypic measurement, sheep eye muscle, ultrasound images, smart livestock management

## Abstract

Accurate EMA assessment in live sheep is crucial for genetic breeding and production management within the meat sheep industry. However, the segmentation accuracy and reliability of existing automated methods are limited by challenges inherent to B-mode ultrasound images, such as low contrast and noise interference. To address these challenges, we present MPG-SwinUMamba, a novel deep learning-based segmentation network. This model uniquely combines the state-space model with a U-Net architecture. It also integrates an edge-enhancement multi-scale attention module (MSEE) and a pyramid attention refinement module (PARM) to improve the detection of indistinct boundaries and better capture global context. The global context aggregation decoder (GCAD) is employed to precisely reconstruct the segmentation mask, enabling automated measurement of the EMA. Compared to 12 other leading segmentation models, MPG-SwinUMamba achieved superior performance, with an intersection-over-union of 91.62% and a Dice similarity coefficient of 95.54%. Additionally, automated measurements show excellent agreement with expert manual assessments (correlation coefficient r = 0.9637), with a mean absolute percentage error of only 4.05%. This method offers non-invasive and efficient and objective evaluation of carcass performance in live sheep, with the potential to reduce measurement costs and enhance breeding efficiency.

## Simple Summary

The eye muscle area (EMA) is a critical indicator for assessing sheep carcass yield and meat quality. To enable precise and non-invasive EMA measurement in live animals, we developed MPG-SwinUMamba, an efficient deep learning segmentation model that automatically and accurately analyzes ultrasound images. Experimental results validated the model’s accuracy and high performance. This automated approach simplifies the data acquisition and analysis pipeline, enhances measurement efficiency and reliability, and provides valuable methodological support for genetic breeding and precision management in the meat sheep industry.

## 1. Introduction

In the intensive production and genetic breeding systems of the modern meat sheep industry, intramuscular fat (IMF) content is a key indicator of meat quality, influencing flavor, tenderness, and juiciness. Accurate assessment of IMF is therefore critical for carcass quality evaluation, meat yield prediction, and economic profitability [1,2]. Eye Muscle Area (EMA) refers to the cross-sectional area of the Longissimus dorsi muscle in sheep. Previous studies have demonstrated a significant correlation between IMF content and EMA, and have shown that EMA is closely associated with carcass lean meat yield, carcass composition, and overall carcass quality in small ruminants [3]. EMA is influenced by factors such as genetic background, nutritional level, age and growth stage, and management conditions, and is therefore widely used as a key indicator when evaluating meat yield and meat quality potential. Thus, a non-invasive, precise, and efficient method for measuring EMA in live sheep is crucial for the early selection of breeding candidates with superior carcass yield and meat quality potential, the optimization of feeding and management strategies, and the enhancement of overall industry value [4,5].

Traditional EMA measurement relies on post-slaughter carcass evaluation, which prevents the phenotype from being obtained directly on breeding candidates. This destructive approach means that high-performing individuals are sacrificed for data and cannot be retained for breeding, significantly limiting breeding progress and industry efficiency [6]. Consequently, the development of efficient and reliable non-invasive detection technologies for live sheep (typically measured during the finishing phase, e.g., 6–12 months of age) has become a pressing industry priority. B-mode ultrasound imaging has emerged as the mainstream technology for in vivo EMA measurement due to its advantages: it is non-invasive, radiation-free, portable, and cost-effective, finding wide application in smart livestock management [7,8,9]. However, B-mode ultrasound is constrained by inherent image quality issues, such as low signal-to-noise ratios, speckle noise, and minimal acoustic impedance differentiation between tissues. These factors result in blurred muscle boundaries, low contrast, and potential artifacts [10,11]. As a result, achieving precise segmentation is highly challenging and remains heavily dependent on operator experience, which severely compromises the objectivity and consistency of the assessment.

Deep learning-based segmentation methods, such as U-Net, have been widely applied in medical imaging [12,13,14,15]. A growing body of literature has examined the expanding and deepening application of deep learning in smart livestock management, with its technology now being extensively applied to key areas such as meat quality assessment, body parameter extraction, and production management [16,17,18,19,20]. For instance, Yang et al. [21] used the U-Net architecture as the first stage of their EIMFS (Estimating Intramuscular Fat in Sheep) model to segment the longissimus dorsi muscle region in sheep ultrasound images. Cao et al. [22] employed deep learning models, including Fully Convolutional Networks and various U-Net variants, for segmenting the eye muscle area in sheep CT (Computed Tomography) images, proving their superior performance. Despite these advances, existing models exhibit clear limitations when applied to sheep ultrasound imagery. Specifically, when dealing with low-contrast eye muscle images characterized by significant artifacts, these models are prone to segmentation leakage or overflow. This drawback makes it difficult to support stable automated measurement and subsequent genetic evaluation [23,24]. Architectures based on Convolutional Neural Network (CNN) are constrained by local receptive fields, struggling to model long-range contextual dependencies. While Vision Transformer (ViT) can capture global information, their quadratic computational complexity is inefficient for high-resolution image processing. They also rely heavily on large-scale datasets and are prone to overfitting in limited-sample animal ultrasound scenarios [25,26]. Although emerging methods like State Space Model (SSM) show potential, they are often directly migrated from other domains. They have not been systematically optimized for the specific characteristics of ultrasound images, such as weak edges and high noise, which limits their performance on this task. These inadequacies lead to limited segmentation accuracy. They also result in low reliability for eye muscle boundaries in complex ultrasound images. Together, these limitations reduce the ability of existing models to accurately identify and consistently delineate the eye-muscle boundaries in noisy and low-contrast ultrasound images. In response to these inadequacies, we propose a deep learning segmentation network named MPG-SwinUMamba, designed for the high-precision segmentation and automated area measurement of sheep eye muscle ultrasound images.

The principal objectives of this study were as follows: (1) to develop a Swin-UMamba-based network integrating Multi-Scale Edge Enhancement, Pyramid Attention Refinement, and Global Context Aggregation Decoder for robust ultrasound segmentation; (2) to establish an automated analysis pipeline extending from segmentation to phenotypic measurement to ensure application-level consistency; and (3) to validate the method’s superiority over 12 state-of-the-art models, achieving 91.62% IoU and 4.05% MAPE on a dataset of 710 live sheep images.

## 2. Materials and Methods

### 2.1. Overall Process

**Figure 1:**
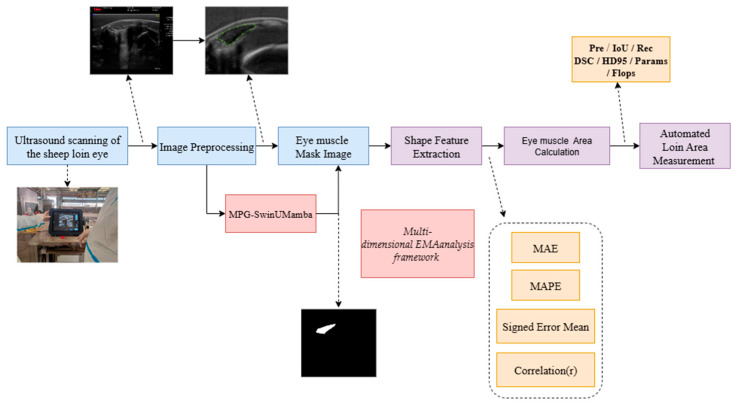
Overall process of the proposed method.

Figure 1 illustrates the automated EMA measurement workflow of our study, including four main steps. First, ultrasound images of live meat sheep are scanned and collected using specialized ultrasound equipment. Second, the collected eye muscle images undergo preprocessing, such as cropping and scaling, and are then input into the trained MPG-SwinUMamba segmentation model to obtain high-precision eye muscle region mask images. Third, the total number of internal pixels is converted into the actual physical area based on calibration. Fourth, the model’s automated physical area measurement is quantitatively compared with the “Ground Truth” area. The Ground Truth values were obtained by experts through the use of scanning equipment and by applying the spatial calibration parameters of the equipment (${mm}^{2}$). This utilizes a multi-dimensional analysis framework that evaluates the method’s consistency and accuracy through metrics such as mean absolute error and correlation coefficient.

### 2.2. Data Acquisition

The meat sheep used in this study were sourced from Qinghuan Meat Sheep Breeding Co., Ltd. in Huan County, Qingyang City, Gansu Province. All sheep were housed in ground pens with ad libitum access to feed and water, following the recommended environmental and nutritional standards (Nutrient Requirements of Meat Sheep, China, NY/T 816-2021 [27]). For in vivo EMA image acquisition, we used the ExaPad mini portable ultrasound scanner (IMV Technologies, Angoulême, France, Serial No. 2503PM03) equipped with a linear array probe. To ensure standardized image quality, the imaging parameters were strictly set as follows: frequency of 2.5 MHz, depth of 160 mm, focal length of 50 mm, dynamic range of 60 dB, and gain of 80. The system operated on the MeatQ software (Humeco, Huesca, Spain, Version 1.6.4). Regarding data collection, images were captured as static screenshots directly from the device upon freezing the optimal cross-sectional view of the eye muscle between the 12th and 13th ribs. This method avoids the motion blur often associated with video frame extraction. The images were saved in JPEG format with a resolution of 730 × 660 pixels.

**Figure 2:**
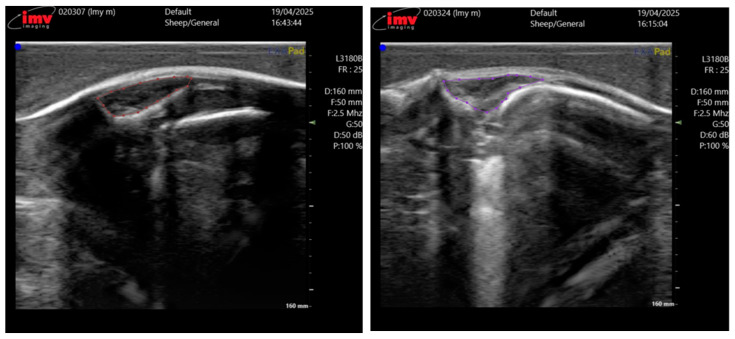
Annotation results for sheep eye muscle ultrasound images. The circled area corresponds to the region of the EMA.

The ultrasound images were acquired by two professional technicians. Both operators were strictly trained to follow the same standard scanning procedure to minimize inter-operator variability. The collected data is uniformly stored and transmitted on the hard drive. We collected ultrasound images from a total of 230 Taonan Lake hybrid sheep, all of which were less than 12 months old, amounting to 710 images, each with a resolution of 730 × 660 pixels. These images were randomly split into training, validation, and test sets at a 7:2:1 ratio, forming the dataset for our eye muscle segmentation experiments. The entire data annotation was performed in a two-stage process to ensure accuracy. First, doctoral students specializing in animal science, trained in ultrasound anatomy, used the LabelMe software 3.16.2 to delineate the eye muscle regions in all 710 images. Subsequently, the annotations were rigorously reviewed and corrected by technical specialists from the device manufacturer and chief domain experts, each possessing over 20 years of research experience in sheep breeding and livestock husbandry, to serve as the ground truth. Figure 2 illustrates the annotation results for two representative meat sheep.

### 2.3. Data Preprocessing

The original high-resolution ultrasound images contained large background regions irrelevant to the eye muscle. This irrelevant information can interfere with model recognition while significantly increasing memory consumption and training time. Therefore, we uniformly resized all input images to 512 × 512 pixels, retaining only the core region containing the eye muscle. To ensure model robustness and reduce the risk of overfitting, we adopted a data augmentation strategy similar to that of nnU-Net [28,29], which primarily included geometric and intensity transformations. Geometric transformations consisted of random rotations, random scaling, and elastic deformations. These operations help the model learn invariance to object shape and orientation, which is particularly important in medical image analysis [13,30,31]. For intensity transformations, we applied random brightness and contrast adjustments and introduced Gaussian noise to simulate random noise interference during the imaging process and help the model adapt to different image qualities. All data augmentation was performed during training, with a certain probability applied to each transformation to ensure diversity in the data. During testing and validation, no data augmentation was used; instead, the images were simply resized to 512 × 512 pixels before being fed into the model for evaluation.

### 2.4. MPG-SwinUMamba Segmentation Model

**Figure 3:**
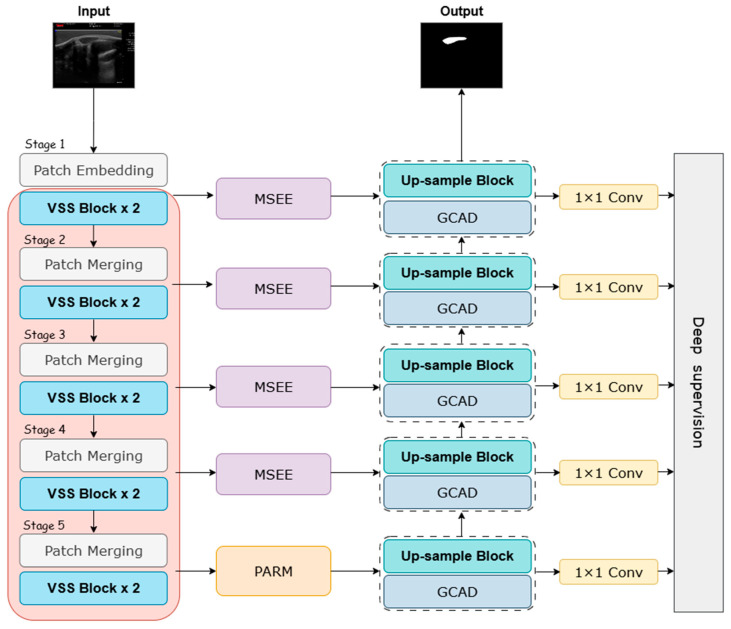
MPG-SwinUMamba Framework.

To systematically address the challenges common in sheep eye muscle ultrasound—such as low contrast, speckle noise, and blurred boundaries—we propose a novel deep learning segmentation network named MPG-SwinUMamba. As shown in Figure 3, this model is an enhanced U-shaped encoder–decoder of Swin-UMamba [32]. We integrated three specialized, synergistic modules: the Multi-scale Edge-Enhanced module, the Pyramid Attention Refinement Module, and the Global Context Aggregation Decoder. This design aims to enhance the model’s ability to delineate ambiguous boundaries and comprehend global context.

Our encoder utilizes an ImageNet pre-trained Swin-UMamba backbone. This encoder leverages hierarchical Visual State Space (VSS) blocks to efficiently capture long-range dependencies within the feature pyramid. At the network bottleneck, where semantic information is richest, the PARM refines and reinforces global morphological priors through parallel pyramid pooling, ensuring a comprehensive understanding of the eye muscle’s overall structure. To address the core problem of weak boundary signals, the MSEE is integrated into the skip connections, fusing traditional edge detection operators with an attention mechanism. This significantly enhances the expression of boundary features and provides the decoder with highly edge-aware feature maps. Finally, the GCAD intelligently merges edge details reinforced by the encoder, global context passed through the bottleneck layer, and its own deep semantic information during the progressive upsampling process. Through the gradual refinement and synthesis of multi-source information streams, the decoder accurately reconstructs the final segmentation result, which balances global consistency with clear local contours.

#### 2.4.1. Efficient Feature Encoder Based on State Space Model

**Figure 4:**
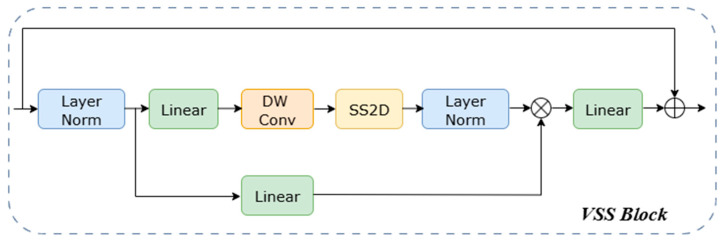
Architecture of VSS Block.

Our encoder employs a five-stage hierarchical structure designed to build a comprehensive feature pyramid. It captures features ranging from local eye muscle details to global structures by progressively decreasing spatial resolution while systematically increasing the channel count from 48 to 768. The process begins with a stem stage, which uses a 7 × 7 large-kernel convolution for 2× downsampling, followed by 2D Instance Normalization. From the second stage onward, the network performs downsampling using patch embedding or patch merging layers, followed by a series of VSS blocks for high-level feature extraction. The number of VSS blocks in these stages is {2, 2, 2, 2, 2}. We use the VSS block as the fundamental unit of MPG-SwinUMamba. It is optimized for 2D image data by introducing the Selective 2D Scanning (SS2D) mechanism. This SS2D mechanism overcomes the limitations of 1D SSMs by employing a four-directional scanning strategy, enabling the comprehensive modeling of spatial relationships in all directions. As detailed in the VSS Block Module (Figure 4), the input feature first passes through Layer Normalization and is then split into two parallel branches. In the first branch, the input is processed by a linear layer and an activation function. In the second branch, the input passes through a linear layer, a depth-wise separable convolution, and an activation function before being fed into the 2D-SSM module for further feature extraction. The output of this second branch is then layer-normalized. The two pathways are fused via element-wise multiplication. Finally, a linear layer mixes the fused features, and the result is added to the residual connection to form the VSS block’s output. Additionally, the module integrates a selective state space mechanism that dynamically adjusts the state transition parameters based on the input content. This mechanism enhances key eye muscle structures and suppresses background noise, providing a significant advantage in handling common artifacts and irregular structures in ultrasound images, which allows the model to focus more robustly on the target eye muscle region. Notably, due to the causal nature of the VSS blocks, our encoder does not employ positional embeddings [33]. During model initialization, the VSS blocks and patch merging layers are directly loaded with ImageNet pre-trained weights from VMamba-Tiny, while the patch embedding layers, due to their structural differences, are initialized randomly.

#### 2.4.2. Multi-Scale Edge-Enhanced (MSEE) Module

**Figure 5:**
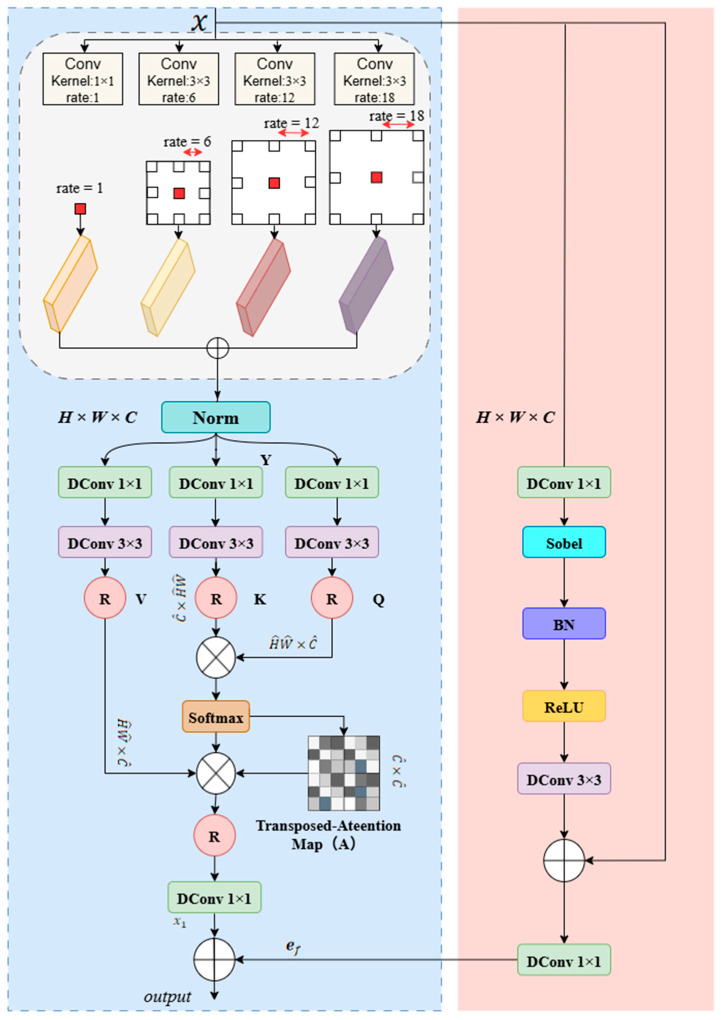
Architecture of MSEE.

To address the inherent limitations of traditional skip connections when handling eye muscle ultrasound images with low signal-to-noise ratios and blurred boundaries, we designed a MSEE. As shown in Figure 5, the module operates with two paths working in parallel: Upon input of image feature *x*, the left path first processes the input through a parallel multi-scale dilated convolution module. This module consists of a 1 × 1 convolution followed by three 3 × 3 dilated convolutions with dilation rates of 6, 12, and 18. The outputs from the four convolutions are summed element-wise to fuse multi-scale contextual information. The fused feature is then processed through a Norm layer and passed through three branches (containing 1 × 1 DConv and 3 × 3 DConv) to generate the query (Q), key (K), and value (V) matrices. After reshaping, the K and Q matrices pass through matrix operations, and a transpose attention map is generated using the Softmax function. This attention map is then multiplied by the reshaped matrices to perform weighted aggregation. Finally, the aggregated feature $x_{1}$ is processed through a DConv layer to produce the output $e_{f}$ from the left path.

The Query (Q), Key (K), and Value (V) are generated by integrating the local context Y with a normalized tensor layer:

(1) $$Q=W_{P}^{Q}W_{D}^{Q}YK=W_{p}^{K}W_{d}^{K}YV=W_{p}^{V}W_{d}^{V}Y$$

The process of the left pathway can be summarized by the following equations:

(2) $$\Lambda =Softmax(\hat{K}⨂\hat{Q})$$

(3) $$e_{f}=D{Conv}_{1\times 1}(R^{-1}\left(\hat{V}⨂\Lambda \right))$$

where $\hat{K}$, $\hat{Q}$, and $\hat{V}$ represent the reshaped matrices, respectively, and $R^{-1}$ represents the inverse reshape operation, and ⨂ represents matrix multiplication.

In the right path, the input feature x first passes through a 1 × 1 DConv layer, followed by a Sobel operator to extract edge information, a Batch Normalization (BN) layer, and a ReLU activation function. The resulting feature map is then processed through a 3 × 3 DConv layer. To retain and enhance the original spatial information, the output of this convolutional layer is combined with the original input feature x through a residual connection. The final sum is then passed through a 1 × 1 DConv layer, yielding the output $e_{s}$ of the right path.

(4) $$x_{edge}=D{Conv}_{3\times 3}(ReLU(BN\left(Sobel\left(D{Conv}_{1\times 1}\left(x\right)\right)\right)))$$

(5) $$e_{s}=D{Conv}_{1\times 1}(x+x_{edge})$$

Finally, the MSEE combines the context features $e_{f}$ from the left path with the edge spatial features $e_{s}$ from the right path by performing element-wise addition, resulting in the module’s final output, output. This fusion creates a powerful complementary effect: the context information provides semantic reference for the blurred edges, while the clear edges offer spatial anchors for the context features. This design transforms the skip connection into an intelligent processing unit, capable of outputting optimized features that balance rich semantics with precise localization. This significantly enhances the model’s segmentation accuracy and robustness under noise and blurring interference, making it a key component for achieving accurate eye muscle area measurement.

#### 2.4.3. Pyramidal Attention Refinement Module (PARM)

**Figure 6:**
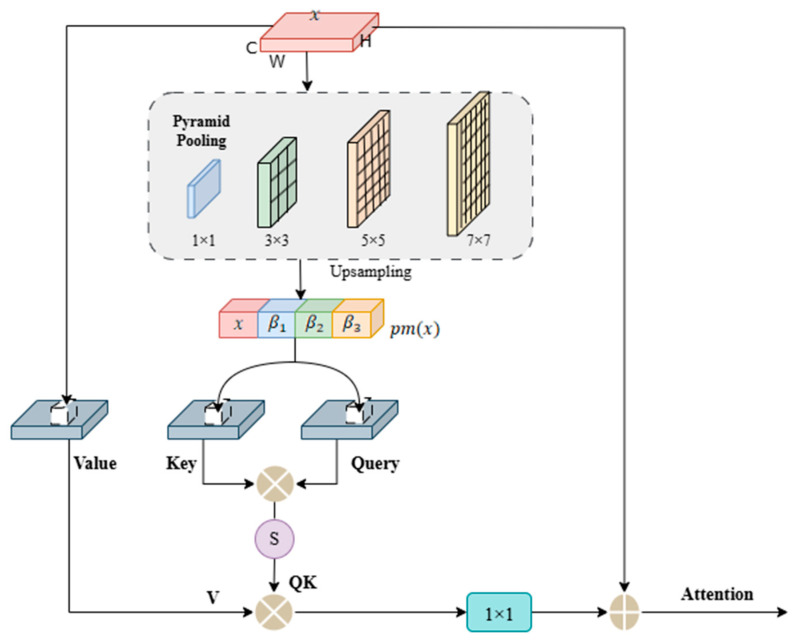
Architecture of PARM.

The bottleneck layer in the network serves as a critical junction between the encoder and decoder, where the model forms the highest-level semantic understanding of the eye muscle target. However, when the encoder extracts high-level features through successive local operations, such as convolution and downsampling, it can lead to a blurred perception of the target’s complete shape. Additionally, the eye muscle in ultrasound images often exhibits high variability in form, size, and spatial distribution, making it challenging for single-scale deep features to robustly capture such complexity. Inspired by Jain D et al. [34], we introduce a PARM at the bottleneck layer. As shown in Figure 6, this module simultaneously performs multi-scale context aggregation and long-range spatial dependency modeling, capturing both the overall contour and internal structure of the eye muscle. This results in an enhanced feature representation with global shape awareness. The features output by the PARM possess both rich semantic and structural awareness, providing crucial guidance to the decoder for accurate boundary reconstruction and improved segmentation consistency.

The PARM operates through an efficient two-stage process. The first stage involves multi-scale pyramid pooling, where multiple parallel pooling branches of different sizes (e.g., 1 × 1, 3 × 3, 5 × 5, 7 × 7) are used to capture the contextual features of the eye muscle at different receptive fields, ranging from global descriptors to local region patterns, forming a compact feature pyramid. The second stage introduces a non-local attention mechanism to explicitly model the intrinsic relationships between multi-scale features. This mechanism computes the similarity between all spatial position pairs, constructing a dense attention matrix that establishes global dependencies across the entire eye muscle region. This allows the model to interpret the eye muscle area as a coherent whole. To ensure the computational efficiency of this powerful mechanism, we integrated optimization strategies. By performing channel compression and grouped processing before the attention calculation, we significantly reduced the computational burden, thereby ensuring the model’s practical utility for real-world applications.

The output feature of the network’s bottleneck layer is denoted as $x\in R^{C\times H\times W}$, where H×W represents the spatial resolution, and C is the number of channels. Before computing the embedded keys and queries, we introduce pyramid pooling. Multi-scale features are obtained through pooling operations of four different sizes (1 × 1, 3 × 3, 5 × 5, 7 × 7). The multi-scale feature maps are upsampled to the original resolution and then concatenated. The algorithm for this process can be represented as follows:

(6) $$pm\left(x\right)=Concat(\beta_{1},\beta_{2},\beta_{3},x)$$

where $pm\left(x\right)$ denotes the pyramid pooling operation, and $\beta_{1}$, $\beta_{2}$, and $\beta_{3}$ represent the features of different sizes obtained after upsampling. Concat(⋅) refers to the concatenation of these upsampled features.

Convolutional layers ϕ(⋅), θ(⋅), and g(⋅) are applied to the concatenated context features after channel compression to reduce computational cost and obtain the query (Q), key (K), and value (V) embeddings.

(7) query=φ(pm(x))

(8) key=θ(pm(x))

(9) value=g(x)

Next, we apply the mappings $W_{j}^{K}(\mathrm{key})$ and $W_{i}^{Q}(\mathrm{query})$ to compress the concatenated context channels, where the shape of the key is $W_{j}^{K}(\mathrm{key})\in R^{C^{\prime }\times H\times W}$ and the query is $W_{i}^{Q}(\mathrm{query})\in R^{C^{\prime }\times H\times W}$. Then, they are multiplied to compute their similarity. QK is normalized by softmax.

(10) $$QK=softmax(W_{j}^{K}(key)⊗W_{j}^{Q}(query))$$

where ⊗ represents matrix multiplication. $W_{j}^{K}(key)$ represents the reshape operation that transposed $\mathrm{value}\in R^{C^{\prime }\times H\times W}$ into $W_{j}^{V}(value)\in R^{{(H\times W)\times C}^{\prime }}$. Then, we multiply QK by V to generate the weighted aggregated matrix. This is followed by a 1 × 1 convolution and the addition of the input feature x. The final output feature map Attention, which incorporates long-range dependencies, is expressed as:

(11) $$V=W_{j}^{V}(value)$$

(12) $$Attention={Conv}_{1\times 1}\left(\Gamma \left(QK⊗V\right)\right)+x$$

where Γ(⋅) represents the reshape operation projecting the features to a size of $C^{\prime }\times H\times W$, ${Conv}_{1\times 1}\left(\cdot \right)$ represents a 1 × 1 convolution, and x is the input feature map.

#### 2.4.4. Global Context Aggregation Decoder (GCAD)

**Figure 7:**
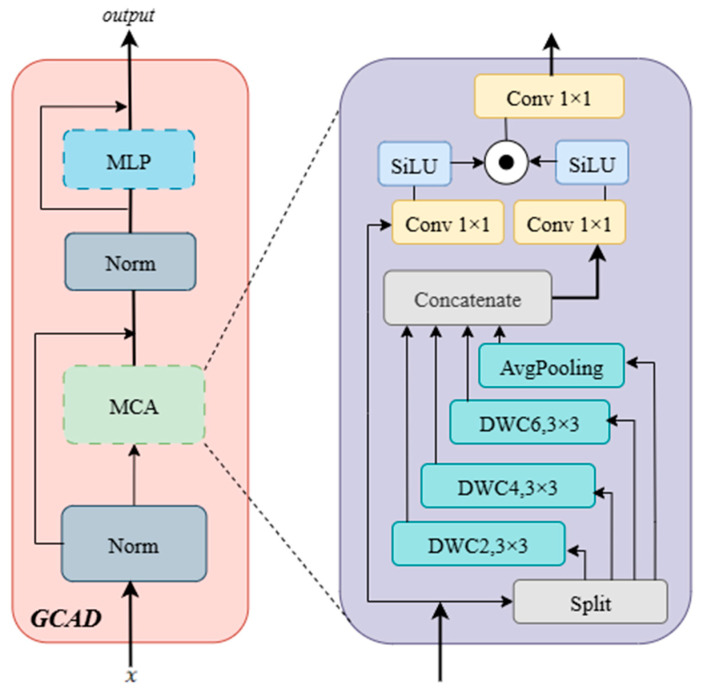
Architecture of GCAD Module.

The core task of the GCAD is to reconstruct an accurate pixel-level eye muscle segmentation map by efficiently integrating and upsampling the hierarchical features extracted by the encoder. To address the challenges of blurred eye muscle boundaries and rich details, our design focuses on progressively restoring resolution while adaptively merging features from different levels through a powerful feature refinement module. This approach optimizes the global guidance of high-level semantics and the local precision of shallow details. As shown in Figure 7, specifically, the input feature X first passes through a normalization (Norm) layer. The output, $X_{A}$, is then fed into the Multi-scale Context Aggregation (MCA) sub-layer. The output of the MCA is processed by a second Norm layer to obtain $X_{B}$. $X_{B}$ and the MCA input $X_{A}$ are then added through a residual connection to produce $X_{S1}$. This is subsequently passed through a multi-layer perceptron (MLP) layer. The output of the MLP is then added to the output of the previous MLP layer through a residual connection, resulting in the final output Y of the GCAD module.

(13) $$X_{S1}=X_{A}+Norm(MCA\left(X_{A}\right))$$

(14) $$Y=X_{S1}+MLP(X_{S1})$$

Inside the MCA sub-layer, the input $X_{A}$ is passed into a multi-branch structure to extract multi-scale information. This structure consists of an average pooling layer (AvgPooling) and three 3 × 3 depthwise separable convolutions with different dilation rates (DWC2, DWC4, DWC6). These branches use a hierarchical fusion approach: the output F1 of DWC6 is added to $X_{A}$ and passed as input to DWC4. The output F2 of DWC4 is added to $X_{A}$ and passed as input to DWC2, producing F3. Simultaneously, the output F1 of DWC6 is added to the output F4 of the average pooling layer. Next, the original input $X_{A}$, the features F2, F3, and the sum of F1 and F4 are concatenated together to form the final feature set.

(15) $$F_{1}={DWC}_{6,3\times 3}(X_{A})$$

(16) $$F_{2}={DWC}_{4,3\times 3}(X_{A}+F_{1})$$

(17) $$F_{3}={DWC}_{2,3\times 3}(X_{A}+F_{2})$$

(18) $$F_{4}=AvgPooling(X_{A})$$

(19) $$X_{con}=Concatenate(X_{A},F_{2},F_{3},(F_{1}+F_{4}))$$

The concatenated features $X_{\mathrm{con}}$ are passed into a gating mechanism, where they are processed in parallel through two 1 × 1 convolutions followed by the SiLU activation function. The outputs of these convolutions, $P_{A}$ and $P_{B}$, are then multiplied element-wise. The result of this multiplication is passed through another 1 × 1 convolution, producing the output $X_{\mathrm{MCA}}$ of the MCA sub-layer.

(20) $$X_{MCA}={Conv}_{1\times 1}(SiLU\left({Conv}_{1\times 1}\left(X_{con}\right)\right)⊙SiLU({Conv}_{1\times 1}\left(X_{con}\right)))$$

#### 2.4.5. Loss Function

To optimize the model for precise sheep eye muscle image segmentation, we have designed a hybrid loss function that combines Dice Loss and Binary Cross-Entropy (BCE) loss. This combination aims to address inherent challenges in medical segmentation, such as class imbalance between foreground anatomical structures and background regions, while ensuring accurate boundary delineation.

Let ${\hat{p}}_{i}$ represent the predicted probability of pixel i, and $g_{i}$ represent the true label, where i∈[1,N] and N is the total number of pixels in the image [35,36]. The Dice loss is defined as:

(21) $$L_{Dice}=1-\frac{2\sum_{i=1}^{N}g_{i}{\hat{p}}_{i}}{\sum_{i=1}^{N}g_{i}+\sum_{i=1}^{N}{\hat{p}}_{i}}$$

The BCE loss is defined as:

(22) $$L_{BCE}=-\frac{1}{N}\sum_{i=1}^{N}\left[g_{i}\log\left({\hat{p}}_{i}\right)+\left(1-g_{i}\right)\log\left(1-{\hat{p}}_{i}\right)\right]$$

The final loss function is a combination of these two components:

(23) $$L_{total}=L_{Dice}+L_{BCE}$$

### 2.5. The Multi-Dimensional Analysis Framework for EMA Measurement Performance

Although metrics such as IoU and DSC effectively evaluate pixel-level segmentation accuracy, they cannot directly quantify the error in the final measured area value. In practical applications such as smart livestock management, obtaining precise and reliable EMA values is crucial. Therefore, relying solely on segmentation metrics is insufficient for a comprehensive evaluation of the model’s practical value. To address this, we have developed a multi-dimensional analysis framework for evaluating EMA measurement performance. The core task of this framework is to conduct in-depth statistical evaluations of the accuracy, bias, and consistency of the automated measurement results from an application perspective. The framework’s process consists of two main stages. First, morphological features are extracted from the binary mask images output by MPG-SwinUMamba. The eye muscle segmentation results from the MPG-SwinUMamba model are represented as binary mask images, where foreground pixels correspond to the EMA, and background pixels represent non-eye muscle areas. The automated measurement of the EMA is obtained by calculating the total number of foreground pixels in the mask image, and then converting this number into the actual physical area using calibration data from the ultrasound device, which provides the physical size each pixel represents.

Next, a set of multi-dimensional statistical metrics is employed to quantitatively compare the automated measurement values with the standard values manually delineated by domain experts. In the context of live sheep genetic breeding and production practices, deploying an accurate and automated measurement method offers significant advantages: (1) non-invasive measurement, enabling continuous monitoring of breeding stock growth and development; (2) significantly improved measurement efficiency, making large-scale screening feasible; and (3) elimination of subjective differences caused by manual operations, ensuring the objectivity and repeatability of measurement results.

To achieve a comprehensive and in-depth performance analysis, the framework integrates four complementary core statistical evaluation metrics, replacing a single error measurement. The core metrics of this framework include: MAE and MAPE, which evaluate the absolute accuracy and relative performance of the measurements, respectively; Signed Error Mean (SEM), which diagnoses whether the model exhibits systematic overestimation or underestimation bias; and Correlation Coefficient, which measures the linear consistency between the results of two measurement methods. Through a comprehensive analysis of these metrics, we can systematically verify the performance of the proposed automation method.

### 2.6. Experimental Setup and Evaluation Metrics

MPG-SwinUMamba loads the ImageNet pre-trained weights from VMamba-Tiny, and the parameters for network training are detailed in Table 1. All experiments were conducted on the same experimental platform, with the specific configuration shown in Table 2. The segmentation model was built using PyTorch 2.01, and OpenCV 4.11 was used for preprocessing the raw images and extracting features from the mask images.

In the sheep eye muscle segmentation experiment, several widely recognized evaluation metrics were used to assess the model’s performance. For segmentation performance, the metrics include IoU, DSC, Recall (Rec), Specificity (Spe), Precision (Pre), and 95% Hausdorff Distance (HD95). For model efficiency, we introduced Parameters (Params) to measure model size and Floating-point of Operations (Flops) to measure time complexity (computational load). IoU evaluates the accuracy of segmentation by calculating the ratio of the intersection to the union of the predicted region and the ground truth region. DSC quantifies the degree of overlap between the predicted and true regions. Rec measures the model’s ability to detect the actual eye muscle region or its completeness in covering the target area. Spe reflects the model’s ability to correctly identify background regions and areas outside the eye muscle, thus distinguishing non-eye muscle areas. Pre is a critical metric that evaluates how accurately the model predicts the eye muscle region. HD95 measures the 95th percentile of the Hausdorff distance between the predicted and true boundaries, assessing how closely the segmentation boundaries align with the ground truth. Params (M) is the number of parameters (in millions) representing the model’s spatial complexity and size, and Flops is the number of floating-point operations (in Giga-FLOPs) quantifying the model’s time complexity and computational load. The formulas for calculating IoU, DSC, Rec, Spe, and Pre are as follows:

(24) $$IoU=\frac{TP}{TP+FP+FN}$$

(25) $$DSC=\frac{2TP}{2TP+FP+FN}$$

(26) $$Rec=\frac{TP}{TP+FN}$$

(27) $$Spe=\frac{TN}{TN+FP}$$

(28) $$Pre=\frac{TP}{TP+FP}$$

In these metrics, TP (True Positive) refers to the number of eye muscle pixels correctly predicted as eye muscle pixels, FP (False Positive) is the number of background pixels incorrectly predicted as eye muscle pixels, TN (True Negative) is the number of background pixels correctly predicted as background pixels, and FN (False Negative) is the number of eye muscle pixels incorrectly predicted as background pixels.

In the multi-dimensional analysis of EMA measurement, we aimed to conduct an in-depth statistical evaluation of the reliability of the final area values derived from the segmentation masks. To this end, we selected four core metrics that reveal model performance from different dimensions. The MAE directly reflects the average magnitude of the absolute deviation between the automated measurement and the true value. It provides an intuitive measure of the prediction error in pixels. The MAPE is used to assess the relative error, independent of the actual size of the eye muscle area. This ensures that the evaluation results are comparable across samples of varying sizes. SEM measures the model’s systematic bias by calculating the average prediction error while retaining the sign (positive or negative). This metric is crucial for understanding whether the model systematically overestimates (positive value) or underestimates (negative value) the eye muscle area, which is a key indicator of the reliability of an automated measurement tool. Finally, the Correlation Coefficient (r) measures the linear consistency between the automated measurement values and the expert’s manual measurements, indicating the level of agreement between the two methods.

The formulas for MAE, MAPE, SEM, and r are as follows:

(29) $$MAE=\frac{1}{n}\sum_{i=1}^{n}\left|{\hat{Y}}_{i}-Y_{i}\right|$$

(30) $$MAPE=\frac{1}{n}\sum_{i=1}^{n}\left|\frac{{\hat{Y}}_{i}-Y_{i}}{Y_{i}}\right|\times 100\%$$

(31) $$SEM=\frac{1}{n}\sum_{i=1}^{n}\left({\hat{Y}}_{i}-Y_{i}\right)$$

(32) $$r=\frac{\sum_{i=1}^{n}\left(Y_{i}-\bar{Y}\right)\left({\hat{Y}}_{i}-\bar{\hat{Y}}\right)}{\sqrt{\sum_{i=1}^{n}{\left(Y_{i}-\bar{Y}\right)}^{2}\sum_{i=1}^{n}{\left({\hat{Y}}_{i}-\bar{\hat{Y}}\right)}^{2}}}$$

In the formulas, n represents the total number of samples, $Y_{i}$ is the true eye muscle area value manually measured by the expert for the i-th sample, ${\hat{Y}}_{i}$ is the eye muscle area value predicted by the model for the i-th sample, $\bar{Y}$ is the average of all true eye muscle area values across all samples, and $\bar{\hat{Y}}$ is the average of all predicted eye muscle area values across all samples.

## 3. Results

### 3.1. Comparison of Segmentation Models

To validate the performance of MPG-SwinUMamba, we compared its experimental results against 12 other models: Unet [36], AttUNet [37], UNet++ [38], Unet3+ [39], TransUNet [40], SegResNet [41], nnUNet [42], LightMUNet [43], LKMUNet [44], UmambaBot [45], UmambaEnc [45], and SwinUMamba [32]. The training parameters and loss functions for all competing models were kept consistent with MPG-SwinUMamba.

The segmentation results are detailed in Table 3. MPG-SwinUMamba achieved the best performance among all models, with Pre, IoU, Rec, DSC, and HD95 scores of 95.90%, 91.62%, 95.39%, 95.54%, and 5.62, respectively. In terms of computational efficiency, our model maintains a competitive balance between performance and cost. As shown in Table 3, MPG-SwinUMamba operates with 61.12 M parameters and 176.85 G Flops. While this indicates a moderate computational footprint, it yields the highest segmentation accuracy, outperforming larger models like LKMUNet (109.42 M parameters) and TransUNet (105.32 M parameters) in terms of resource utilization efficiency. Compared to SwinUMamba, which uses the same encoder, our model showed improvements in four of these key metrics. Notably, our model’s IoU was 3.84% higher than that of SwinUMamba, which ranked second in accuracy.

**Figure 8:**
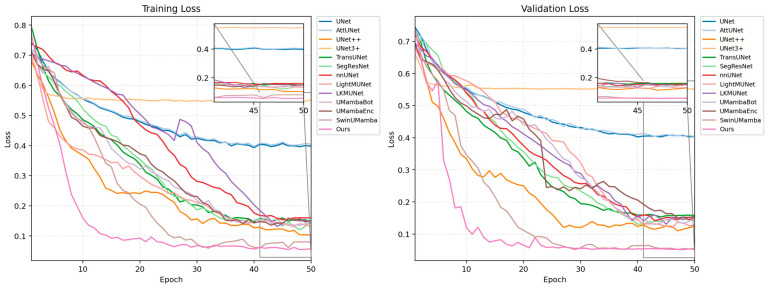
Training and validation loss curves of different segmentation models.

Figure 8 illustrates the loss curves for all models on the training and validation sets. As the number of epochs increased, the loss values for all models decreased and eventually stabilized. The plots show that, compared to the other models, our proposed MPG-SwinUMamba model converges faster during the initial training phase and consistently maintains the lowest loss value. Specifically, MPG-SwinUMamba converged to a lower loss level on the validation set, indicating that our model performs better on unseen data and possesses stronger generalization ability and higher accuracy.

### 3.2. Ablation Experiment

We conducted ablation experiments to validate the effectiveness of our proposed MPG-SwinUMamba model for the automated sheep eye muscle area measurement task. As detailed in Table 4, the experiments involved three model configurations: E0, which used the SwinUMamba architecture with the MSEE; E1, which built upon E0 by adding the PARM to enhance feature extraction; and E2, which built upon E1 by incorporating the GCAD module to optimize the decoding process.

By training the SwinUMamba architecture combined with the MSEE (E0) on eye muscle image data, the IoU value achieved was 88.08%, and the DSC value was 93.57%, indicating that this model has good baseline performance in sheep eye muscle area segmentation. The Recall and Precision values were 93.81% and 93.66%, respectively, showing that MPG-SwinUMamba maintains a high correlation in pixel-level predictions. However, the HD95 value was 9.90 mm, reflecting that there is room for improvement in boundary segmentation accuracy. After adding the PARM to form the enhanced model (E1), the IoU value improved to 90.10%, and the DSC increased to 94.73%, indicating that the introduction of the attention mechanism effectively enhanced feature representation. Additionally, Recall and Precision rose to 94.97% and 94.75%, while HD95 significantly decreased to 7.64 mm, further validating the role of the GPAM module in improving segmentation details and boundary accuracy. When the GCAD module was further introduced to form the final model (E2), the IoU increased to 91.62%, and the DSC reached 95.54%, showing a significant performance improvement. Recall and Precision reached 95.39% and 95.90%, respectively, while HD95 dropped to 5.6157 mm, indicating that feature fusion during the decoding process effectively optimized the coherence and boundary accuracy of the segmentation results. As the model progressed from E0 to E2, the IoU value increased from 88.08% to 91.62%, a 4.02% improvement, and the DSC value increased from 93.57% to 95.54%, a 2.11% increase. Specifically, the baseline model (E0) already exhibited reliable performance, and the model with the added PARM (E1) outperformed E0 across all metrics. The final model (E2), combining both PARM and GCAD, demonstrated the best performance. Therefore, the proposed method outperforms the baseline model in the sheep eye muscle area measurement task, especially after incorporating multi-stage attention and decoding mechanisms. These results validate the effectiveness of progressive module fusion in enhancing segmentation accuracy and robustness.

### 3.3. Automated Eye Muscle Area Measurement Results

To quantitatively evaluate the accuracy of the EMA calculated from the segmentation masks, we applied our “multi-dimensional performance analysis framework for EMA measurement.” The automated measurements from the MPG-SwinUMamba model and other comparison models were compared with the expert’s manual measurements.

Table 5 presents the descriptive statistics of EMA measurements for the entire test dataset. It details the Mean ± Standard Deviation (SD), Minimum, and Maximum values for both the manual-delineated standard (Ground Truth) and the MPG-SwinUMamba automated method. The results demonstrate a high degree of consistency between the two approaches. Notably, the mean value of the automated method (863.85 ${mm}^{2}$) is closely aligned with that of the ground truth (815.04 ${mm}^{2}$), with a minimal systematic difference. This indicates that our model accurately captures the average phenotypic characteristics of the population without significant bias. Furthermore, the close alignment of the minimum and maximum values confirms the model’s robustness across individuals with varying muscle sizes, maintaining stable performance from small to large phenotypes.

Within this analysis framework, the MPG-SwinUMamba model demonstrates exceptional performance. In terms of accuracy, the model’s MAE is as low as 356.89 pixels, and the MAPE is only 4.05%, significantly outperforming other comparison models such as UNet++, where the MAE and MAPE are 864.82 pixels and 9.89%, respectively. This highlights the substantial advantage of our model in terms of prediction accuracy. For bias analysis, our model’s SEM was −72.83 pixels. This is an excellent result compared to models like TransUNet (138.09 pixels), LightMUNet (127.83 pixels), and AttUNet (1609.67 pixels), as a value close to zero indicates an unbiased prediction. Furthermore, the automated measurements showed extremely high linear consistency with the expert-delineated standard values, achieving a correlation coefficient of 0.9637. This strongly proves the reliability of our automated measurement method.

## 4. Discussion

### 4.1. Discussion of Data Processing

We processed the original high-resolution ultrasound images by cropping and uniformly scaling them to a 512 × 512 resolution. This step was essential for removing irrelevant background regions and directing the model’s focus to the eye muscle. Concurrently, data augmentation strategies, such as geometric and intensity transformations, were applied during the training phase. These strategies significantly enhanced the model’s robustness against various artifacts and interferences encountered during practical acquisition, thereby improving its generalization capability. Although image downsampling may result in the loss of some fine tissue texture information, it enables the model to train efficiently and perform rapid predictions using limited computational resources. This trade-off is critical for the technology’s promotion and deployment in practical farming environments. The experimental results confirmed that our image data processing strategy is effective. This approach strikes a good balance between theoretical performance and practical application, maintaining segmentation accuracy while ensuring training efficiency and deployment feasibility.

### 4.2. Discussion of the Evaluation Metrics

We employed a comprehensive and in-depth analysis framework built on three complementary dimensions: pixel-level accuracy, regional overlap, and boundary fidelity. Pixel-level accuracy was jointly evaluated using Pre and Rec. However, as pixel-level metrics alone cannot fully capture the spatial congruence of the segmentation contour, we introduced IoU and DSC to quantify regional overlap. These metrics provide a more holistic measure of spatial agreement and are more robust to the class imbalance between the eye muscle target and the background. Finally, given that EMA measurement accuracy is highly dependent on contour precision, we used the HD95—a metric highly sensitive to boundary deviations—to directly assess boundary fidelity. This three-dimensional evaluation framework ensures a comprehensive consideration of the model’s segmentation performance. In addition to accuracy, considering the limited computing power of portable ultrasound devices in practical breeding scenarios, we also introduced Parameters (Params) and Floating-point Operations (Flops). These metrics serve to evaluate the model’s spatial and temporal complexity, ensuring that high precision does not come at the cost of deployment feasibility.

Within the multi-dimensional performance analysis framework for EMA measurement, our goal was to statistically validate the reliability of the final area values derived from the segmentation masks. To this end, we evaluated performance across four key dimensions: absolute accuracy, relative performance, systematic bias, and consistency. We employed MAE and MAPE to quantify absolute and relative errors, respectively, allowing for an efficient assessment of relative performance independent of the actual EMA size. We also used the SEM to diagnose systematic Bias [46]—that is, whether the model tends to overestimate or underestimate the area, a critical factor for a reliable automated tool. Finally, the r was used to measure the linear consistency between automated and manual expert measurements. A high correlation indicates that the model’s predictions effectively track the trend of the ground-truth values. By synthesizing these metrics, this analysis framework provides a comprehensive and rigorous validation of our automated EMA measurement method.

### 4.3. Discussion of MPG-SwinUMamba

The MPG-SwinUMamba model demonstrated highly competitive performance in this study. It demonstrated superior quantitative metrics against 12 mainstream and state-of-the-art segmentation models. This performance also reflected the effectiveness of its systematic architectural innovations, specifically designed for the characteristics of sheep eye muscle ultrasound images. Compared to classic CNN architectures like U-Net and AttUNet, our model achieved significant improvements across key metrics, including IoU, DSC, and HD95. This performance advantage likely stems from the model’s successful fusion of SSM with attention mechanisms. This approach effectively overcomes the inherent limitations of traditional CNN, such as restricted receptive fields and difficulty in modeling long-range dependencies. Furthermore, when compared to Vision Transformer-based models like TransUNet, which also focus on global modeling, MPG-SwinUMamba maintains high accuracy while avoiding the quadratic computational complexity associated with high-resolution images. Quantitative analysis confirms this advantage: our model achieves superior DSC (95.54%) with significantly fewer parameters (61.12 M) compared to TransUNet (105.32 M) and LKMUNet (109.42 M). This balance of high precision and controlled computational cost (176.85 G Flops) suggests greater practicality and robustness for deployment on resource-constrained agricultural devices. It also mitigates the tendency to overfit on small datasets, showing greater practicality and robustness. This advantage is reinforced by comparisons with other research. For instance, Yang et al. [21] used a classic U-Net architecture to segment sheep ultrasound images and reported a DSC of 0.916; our model achieved 0.9554. This performance gap is primarily because MPG-SwinUMamba effectively captures global dependencies via its SSM backbone and PARM, overcoming U-Net’s local constraints. Concurrently, our MSEE is specifically designed to enhance the blurred contours characteristic of ultrasound’s weak boundaries and high noise—a feature standard architectures lack. Moreover, Cao et al. [22] achieved a DSC of 0.947 using an Attention-UNet on sheep CT images, which typically have less noise and higher contrast. Our model outperformed this result on more challenging ultrasound data, highlighting its robust adaptability to complex imaging conditions.

The effectiveness of each module was validated through systematic ablation experiments. Integrating the PARM (E1 model) significantly reduced the HD95 metric from 9.90 mm (baseline E0) to 7.64 mm. This confirms that introducing multi-scale context aggregation and non-local attention at the bottleneck layer greatly enhances the model’s perception of the overall eye muscle morphology and improves segmentation consistency. The final E2 model, leveraging GCAD, further optimized the HD95 to 5.62 mm, illustrating the decoder’s critical role in intelligently fusing deep global semantics with reinforced shallow edge features. This progressive performance improvement validates the synergistic effectiveness of the modules: PARM builds global morphological understanding, MSEE focuses on boundary signal enhancement, and GCAD excels at detail reconstruction and information fusion.

In summary, the exceptional performance of MPG-SwinUMamba validates the effectiveness of its hybrid architecture, which was specifically designed for the characteristics of ultrasound images. By deeply integrating global semantic modeling, local edge enhancement, and multi-scale feature fusion, the model showcased its advanced performance on the challenging task of sheep eye muscle segmentation. It also provides a valuable design paradigm and technical framework for addressing similar low-SNR biomedical image segmentation problems.

### 4.4. Discussion of the EMA Multi-Dimensional Analysis Framework

The EMA multi-dimensional performance analysis framework developed in this study enables a deep interpretation of automated measurement results by integrating four evaluation dimensions: MAE, MAPE, SEM, and r. This approach surpasses the limitations of traditional methods that rely solely on segmentation overlap metrics like Dice. The framework revealed that the MPG-SwinUMamba model, with a MAPE of 4.05% and an MAE of 356.89 pixels, significantly outperformed competing models such as UNet++ (MAPE = 9.89%), indicating stable measurement accuracy across individuals of different sizes. Critically, our model exhibited an extremely low SEM of −72.83 pixels, approaching an unbiased estimate. In contrast, models like AttUNet showed significant overestimation (SEM = 1609.67). This discrepancy likely stems from its attention mechanism’s over-sensitivity to the noise and artifacts in ultrasound images, leading to boundary misjudgments. By comparison, our model’s MSEE and context-aware mechanisms effectively suppressed this interference, thus achieving an unbiased estimate. Van Erck et al. [47] reported an Intraclass Correlation Coefficient (ICC) of 0.96 for automated manual psoas major muscle measurement using CT images. While we acknowledge that r primarily measures linear correlation, our model demonstrated a negligible systematic bias (SEM = −72.83 pixels) in the prior analysis. This interpretation is supported by methodological principles in livestock ultrasound phenotyping, which suggest that correlation coefficients alone are of limited value for evaluating accuracy without a concurrent assessment of prediction error components. As highlighted by Esquivelzeta et al. [48], a rigorous validation requires decomposing the error to ensure that the measurement trueness is not compromised by the Error of Central Tendency (systematic bias), thereby confirming that a high correlation reflects genuine accuracy rather than merely a linear trend. Furthermore, our results satisfy the validation standards recommended by the International Committee for Animal Recording. As detailed by Kruk et al. [49], a reliable measurement method must demonstrate a minimal difference between the scan and the ground truth, alongside a correlation coefficient of at least 0.82. This strong correlation not only validates the high linear consistency between automated results and expert annotations but also demonstrates that the model can reliably track true variations in area. This provides critical technical support for genetic evaluations in breeding practices that rely on the precise ranking of individuals by EMA. Practically, this framework could be integrated into portable ultrasound systems. This would enable veterinarians and technicians to obtain real-time, objective EMA measurements directly on-pasture, facilitating immediate data-driven decisions for genetic selection and herd management. Looking ahead, expanding this system for multi-site or multi-season rollout (e.g., across different sheep types, environments) will be our future focus. This presents challenges, as data variations may introduce correlated errors. As highlighted by Zhao et al. [50], robust strategies like dual-layer filtering are required to maintain stable and accurate predictions in such diverse contexts. We will investigate these methods in future development.

Furthermore, by quantitatively assessing absolute accuracy, relative performance, systematic bias, and consistency, our multi-dimensional analysis framework systematically validates the high reliability of the MPG-SwinUMamba automated measurement method. This framework not only confirms our method’s robust performance under challenging imaging conditions but also provides a generalizable and rigorous paradigm for evaluating automated image-based phenotyping in the livestock industry, guiding future optimization and large-scale application.

## 5. Conclusions

We designed and validated a novel deep learning model, MPG-SwinUMamba, to address the technical challenges of segmenting and automatically measuring in vivo sheep eye muscle ultrasound images. By effectively fusing SSMs with attention mechanisms, the model synergistically extracts global contextual information and local edge features. This approach significantly enhances segmentation accuracy and robustness, particularly on low-quality ultrasound images. Experimental results demonstrated that MPG-SwinUMamba outperformed 12 competing models across key metrics, including Precision, IoU, and DSC, supporting its potential for automated in vivo phenotyping.

This study contributes to both the development of a high-performance segmentation architecture and the systematic validation of a novel, deep-learning-driven phenotyping paradigm for smart livestock farming. Our findings demonstrate that by integrating advanced vision models with domain-specific knowledge, we can achieve measurement accuracy comparable to manual methods, even on challenging images with high noise and weak boundaries. This provides critical support for efficient, non-invasive phenotyping in breeding and offers a methodological framework for transitioning the industry from traditional judgment toward data-driven decision-making.

Furthermore, this study implies the importance of a comprehensive assessment of segmentation accuracy, systematic bias, and linear consistency for fully judging an automated tool’s practical value. This multi-dimensional evaluation framework provides a reference for establishing more rigorous evaluation criteria in related research. Although the model performed exceptionally well on our dataset, its generalization capability requires further validation in broader populations and under varied imaging conditions. However, as the scope of this study was limited to ultrasound data from a specific sheep breed, future studies should focus on expanding validation in multi-center and multi-sample environments to ensure generalization, and on integrating this efficient model into portable devices to facilitate the widespread adoption of precision livestock technology and contribute to the industry’s modernization.

## Figures and Tables

**Table 1:** Training parameters of MPG-SwinUMamba.

Parameters	Value
Epochs	50
Batch size	12
Learning rate	1 × 10^−4^
Optimizer	AdamW
Weight decay	5 × 10^−2^

**Table 2:** The configuration of the experimental platform.

Configuration	Parameter
Operating system	Ubuntu 22.04
CPU	Intel Core i7-12700K
GPU	NVIDIA GeForce RTX 4090
Development language	Python 3.10
Framework	PyTorch 2.01 + Mamba 1.4
CUDA version	CUDA 11.8

**Table 3:** Performance of different segmentation models. This indicates that our method shows significant improvement (*p* < 0.05). In “a ± b,” a and b represent the mean and standard deviation of different subjects, respectively.

Model	Pre	IoU	Rec	DSC	HD95 (mm)	Params (M)	Flops (G)
Unet	0.8114 ± 0.074	0.7901 ± 0.048	0.9679 ± 0.046	0.8828 ± 0.040	17.98 ± 0.52	24.89	112.92
AttUNet	0.8182 ± 0.073	0.7939 ± 0.043	0.9640 ± 0.055	0.8851 ± 0.038	18.26 ± 0.34	34.88	133.27
UNet++	0.9141 ± 0.077	0.8374 ± 0.038	0.9090 ± 0.060	0.9115 ± 0.036	14.59 ± 0.28	47.19	400.31
UNet3+	0.8443 ± 0.068	0.8098 ± 0.039	0.9519 ± 0.097	0.8949 ± 0.038	17.67 ± 0.35	26.97	399.86
TransUNet	0.9053 ± 0.056	0.8386 ± 0.040	0.9192 ± 0.086	0.9122 ± 0.036	12.70 ± 0.37	105.32	64.48
SegResNet	0.9266 ± 0.057	0.8713 ± 0.042	0.9387 ± 0.069	0.9303 ± 0.033	11.32 ± 0.41	6.30	62.64
nnUNet	0.9175 ± 0.061	0.8529 ± 0.032	0.9267 ± 0.071	0.9190 ± 0.034	13.28 ± 0.36	46.33	155.24
LightMUNet	0.8962 ± 0.055	0.8219 ± 0.036	0.9116 ± 0.070	0.8988 ± 0.035	19.75 ± 0.24	5.01	8.51
LKMUNet	0.9260 ± 0.046	0.8584 ± 0.029	0.9243 ± 0.065	0.9224 ± 0.041	12.83 ± 0.28	109.42	339.59
UMambaBot	0.9230 ± 0.054	0.8708 ± 0.024	0.9410 ± 0.050	0.9300 ± 0.025	10.93 ± 0.22	86.80	202.02
UMambaEnc	0.9256 ± 0.052	0.8646 ± 0.026	0.9323 ± 0.056	0.9264 ± 0.024	11.91 ± 0.19	92.01	213.28
SwinUMamba	0.9337 ± 0.046	0.8778 ± 0.018	0.9379 ± 0.054	0.9340 ± 0.026	10.20 ± 0.14	59.89	175.74
**Ours**	**0.9590 ± 0.045**	**0.9162 ± 0.016**	**0.9539 ± 0.046**	**0.9554 ± 0.019**	**5.62 ± 0.18**	61.12	176.85

**Table 4:** Comparison of ablation experiments.

Model	MSEE	PARM	GCAD	IoU	DSC	Rec	Pre	HD95(mm)
E0	✓			0.8808 ± 0.023	0.9357 ± 0.022	0.9381 ± 0.048	0.9366 ± 0.047	9.90 ± 0.24
E1	✓	✓		0.9010 ± 0.017	0.9473 ± 0.021	0.9497 ± 0.046	0.9475 ± 0.048	7.64 ± 0.20
E2	✓	✓	✓	0.9162 ± 0.016	0.9554 ± 0.019	0.9539 ± 0.046	0.9590 ± 0.045	5.62 ± 0.18

**Table 5:** Descriptive statistics of EMA measurements.

Measurement Method	Mean ± SD (${mm}^{2}$)	Min (${mm}^{2}$)	Max (${mm}^{2}$)
Manual Delineation (Ground Truth)	815.04 ± 210.48	356.00	1490.00
Automated Method	863.85 ± 209.40	164.74	1843.71
Difference (Bias)	48.81 ± 59.33	/	/

## Data Availability

The code can be requested from the corresponding authors. The data are not publicly available due to being part of an ongoing study and privacy.

## Abbreviations

EMA	Eye Muscle Area
MSEE	Multi-scale Edge-enhanced
PARM	Pyramidal Attention Refinement Module
GCAD	Global Context Aggregation Decoder
IMF	Intramuscular Fat
EIMFS	Estimating intramuscular fat in sheep
CT	Computed Tomography
CNN	Convolutional Neural Network
ViT	Vision Transformer
SSM	State Space Model
IoU	Intersection over Union
DSC	Dice Similarity Coefficient
MAE	Mean Absolute Error
MAPE	Mean Absolute Percentage Error
VSS	Visual State Space
SS2D	Selective 2D Scanning
BN	Batch Normalization
Norm	Normalization
MCA	Multi-scale Context Aggregation
MLP	Multi-Layer Perceptron
BCE	Binary Cross-Entropy
SEM	Signed Error Mean
Rec	Recall
Spe	Specificity
Pre	Precision
HD95	95% Hausdorff Distance
Params	Parameters
Flops	Floating-point of Operations
TP	True Positive
FP	False Positive
TN	True Negative
FN	False Negative
ICC	Intraclass Correlation Coefficient
SD	Standard Deviation
